# Managing Ventricular Wall Rupture as a Complication of Late Presentation MI in a Complex Patient with Ankylosing Spondylitis

**DOI:** 10.1155/2024/3908939

**Published:** 2024-02-15

**Authors:** Waleed Shaker, Ahsan A. Khan, Michael K. O. Boateng, Mahmoud Abdelziz, Lampson Fan

**Affiliations:** ^1^New Cross Hospital, The Royal Wolverhampton NHS Trust, Wolverhampton Road, WV10 0QP Wolverhampton, UK; ^2^University of Birmingham, Edgbaston, Birmingham B15 2TT, UK

## Abstract

Ventricular free wall rupture is an infrequent but serious complication of myocardial infarction with high mortality despite surgical intervention. In recent years with the COVID-19 pandemic, observational studies have reported a rise in this complication most likely due to patient hesitation in seeking urgent medical assistance for fear of contracting COVID-19 in a hospital setting. This case report highlights the early recognition and diagnosis of ventricular wall rupture by the heart team with a good surgical outcome in a complex patient with ankylosing spondylitis. Ventricular rupture should be considered in deteriorating patients presenting with suspicion of late presentation myocardial infarction. Clinicians in the post-COVID-19 era should expect to see these complications more frequently.

## 1. Introduction

Ventricular free wall rupture is an infrequent but serious complication that occurs within the first 96 hours post-MI with mortality rates as high as 88.2% [1]. Cardiac surgery is the mainstay of treatment [2], but despite surgical intervention, patients who undergo successful repair have a 58.9% survival rate after 3 years [3]. In recent years, the COVID-19 pandemic has had a dramatic effect on life in the world and has significantly impacted and burdened the medical community. This is especially concerning for patients who may be suffering from myocardial infarctions, as a delay in treatment can lead to devastating consequences [4, 5]. Since the onset of the COVID-19 pandemic in 2020, there has been a much higher incidence of mechanical complications, such as valvular or ventricular wall ruptures, from myocardial infarctions due to delay in treatment [6, 7] most likely due to patient hesitation in seeking urgent medical assistance for fear of contracting COVID-19 in a hospital setting [8].

## 2. Case

**Figure 1:**
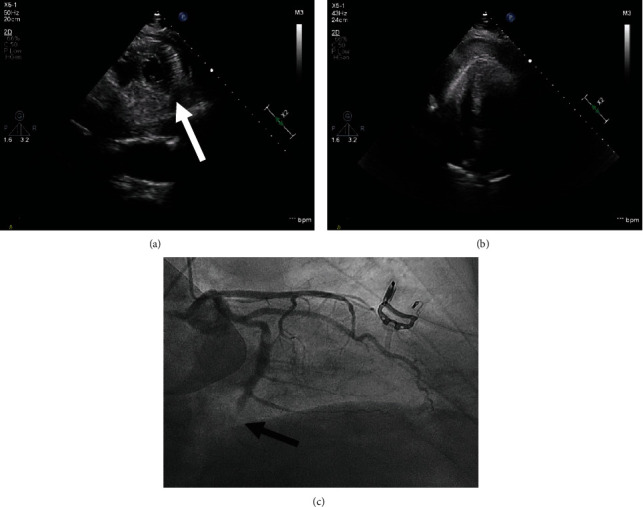
(a) 2D TTE colour Doppler (CFD) echocardiography parasternal short axis (PSAX) view showing LV rupture at the level of the inferior wall with communication between LV and pericardial space (white arrow); (b) 2D TTE apical 4 chamber (A4C) view at presentation showing global large pericardial effusion with clot; (c) coronary angiogram showing occluded dominant distal left circumflex artery (black arrow).

A 54-year-old gentleman was admitted to our hospital following a collapse in public. He had no cardiac history but has a history of ankylosing spondylitis with severe kyphosis. He reported chest pain five days prior to presentation. On arrival at the hospital, he was haemodynamically unstable with a blood pressure of 63/48 mmHg. 12-lead electrocardiogram showed ST-segment elevation in inferior leads with established Q waves consistent with late presenting inferior ST-elevation MI (Supplementary Figure 1). A bedside echocardiogram showed a moderately impaired ventricle with an ejection fraction of 40% and suspected LV wall rupture at the apical inferior wall with a moderate-size pericardial effusion mainly around the LV apex with clots (Figures 1(a) and 1(b); Supplementary video 1-3). A provisional diagnosis of cardiac tamponade due to LV rupture was made. Following discussion with cardiac surgeons, a coronary angiogram was performed which showed an occluded dominant distal left circumflex artery (Figure 1(c)) and confirmed that this is likely an ischaemic ventricular rupture. An intra-aortic balloon pump was inserted in an attempt to stabilize the patient which improved his systolic blood pressure to 90/50 mmHg and brought him sufficient time to have emergency surgery.

**Figure 2:**
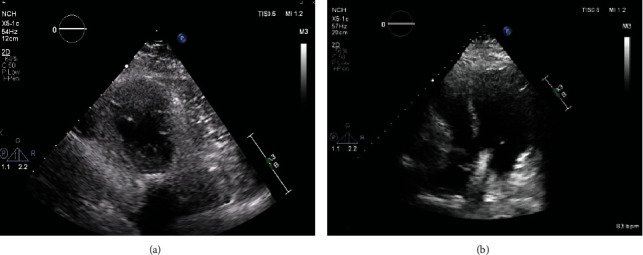
Postsurgery 2D echocardiography. (a) Parasternal short axis view and (b) apical 4 chamber view.

The patient was transferred to the theatre for emergency surgery. He was unable to be intubated due to the severe kyphosis, so cardiopulmonary bypass was established peripherally under local anesthetic with femoral-femoral bypass whilst the patient was awake. Opening of the chest wall revealed a contained pericardial effusion around the LV apex with clots. The LV wall rupture was visualized along with evidence of acute MI laterally. The rupture was successfully repaired on beating heart bypass. Postsurgery TTE confirmed resolution of pericardial effusion and repair of the LV wall (Figure 2; Supplementary Video 4, 5; Supplementary Figure 2). The patient's postoperative course was uneventful, and he was subsequently discharged home. At 10 months, the patient remained well, and his TTE showed EF of 40% with no pericardial effusion.

## 3. Summary

In summary, ventricular rupture should be considered in deteriorating patients presenting with suspicion of late presentation myocardial infarction. Early recognition, diagnosis, and involvement of the surgical team can achieve a good outcome for ventricular rupture especially in complex patients. An early bedside echo was a crucial step in the management of our patient and allowed serial assessment of the LV pre- and postsurgery. Clinicians in the post-COVID-19 era should expect to see these complications more frequently.

## Abbreviations

LV: Left ventricle
MI: Myocardial infarction
TTE: Transthoracic echocardiography
EF: Ejection fraction

## Supplementary Materials

Supplementary materials Figure 1: 12-lead electrocardiogram (ECG) showing left axis deviation, normal sinus rhythm with inferior ST elevation, Q waves, and lateral ST depression. Figure 2: 2D transthoracic echocardiography apical 4 chamber (A4C) view comparison before and after surgery. Supplementary Video 2 A: presurgery apical 4 chamber view. Supplementary Video 2 B: presurgery apical 4 chamber view with colour Doppler. Supplementary Video 2 C: postsurgery apical 4 chamber view. Supplementary Video 2 D: postsurgery apical 4 chamber view with colour Doppler. Video 1: 2D transthoracic echocardiography parasternal short axis (PSAX) view at presentation showing rupture of the left ventricle at the level of the inferior wall. Video 2: 2D transthoracic color Doppler (CFD) echocardiography parasternal short axis (PSAX) view at presentation showing rupture of the left ventricle at the level of the inferior wall with the communication between the left ventricle and pericardial space. Video 3: 2D transthoracic echocardiography apical 4 chamber (A4C) view at presentation showing global large pericardial effusion with organized clot. Video 4: 2D transthoracic echocardiography apical 4 chamber (A4C) LV zoom view postsurgery showing repair of left ventricular wall rupture. Video 5: 2D transthoracic color flow Doppler (CFD) echocardiography apical 2 chamber (A2C) view postsurgery showing repair of left ventricular wall rupture.
