# Supplementary material for: Managing Ventricular Wall Rupture as a Complication of Late Presentation MI in a Complex Patient with Ankylosing Spondylitis
Source: Case Rep Cardiol. 2024 Feb 15;2024:3908939. doi: 10.1155/2024/3908939 (PMC10883737; doi:10.1155/2024/3908939)
Supplement: Supplementary materials — Figure 1: 12-lead electrocardiogram (ECG) showing left axis deviation, normal sinus rhythm with inferior ST elevation, Q waves, and lateral ST depression. Figure 2: 2D transthoracic echocardiography apical 4 chamber (A4C) view comparison before and after surgery. Supplementary Video 2 A: presurgery apical 4 chamber view. Supplementary Video 2 B: presurgery apical 4 chamber view with colour Doppler. Supplementary Video 2 C: postsurgery apical 4 chamber view. Supplementary Video 2 D: postsurgery apical 4 chamber view with colour Doppler. Video 1: 2D transthoracic echocardiography parasternal short axis (PSAX) view at presentation showing rupture of the left ventricle at the level of the inferior wall. Video 2: 2D transthoracic color Doppler (CFD) echocardiography parasternal short axis (PSAX) view at presentation showing rupture of the left ventricle at the level of the inferior wall with the communication between the left ventricle and pericardial space. Video 3: 2D transthoracic echocardiography apical 4 chamber (A4C) view at presentation showing global large pericardial effusion with organized clot. Video 4: 2D transthoracic echocardiography apical 4 chamber (A4C) LV zoom view postsurgery showing repair of left ventricular wall rupture. Video 5: 2D transthoracic color flow Doppler (CFD) echocardiography apical 2 chamber (A2C) view postsurgery showing repair of left ventricular wall rupture. [file 3908939.f1.zip › Supplementary files (1).pptx]

## Slide 1
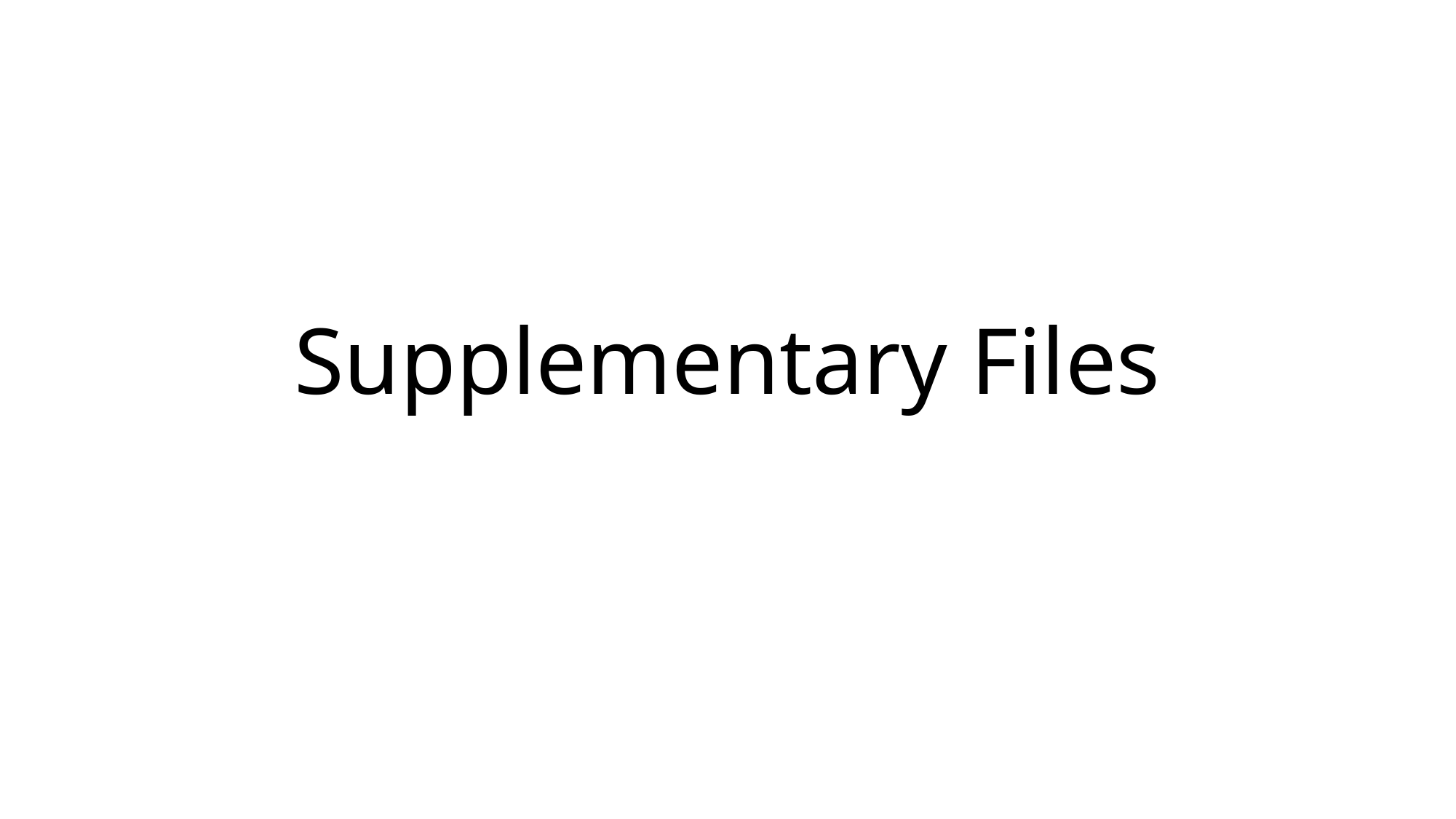

# Supplementary Files

## Slide 2
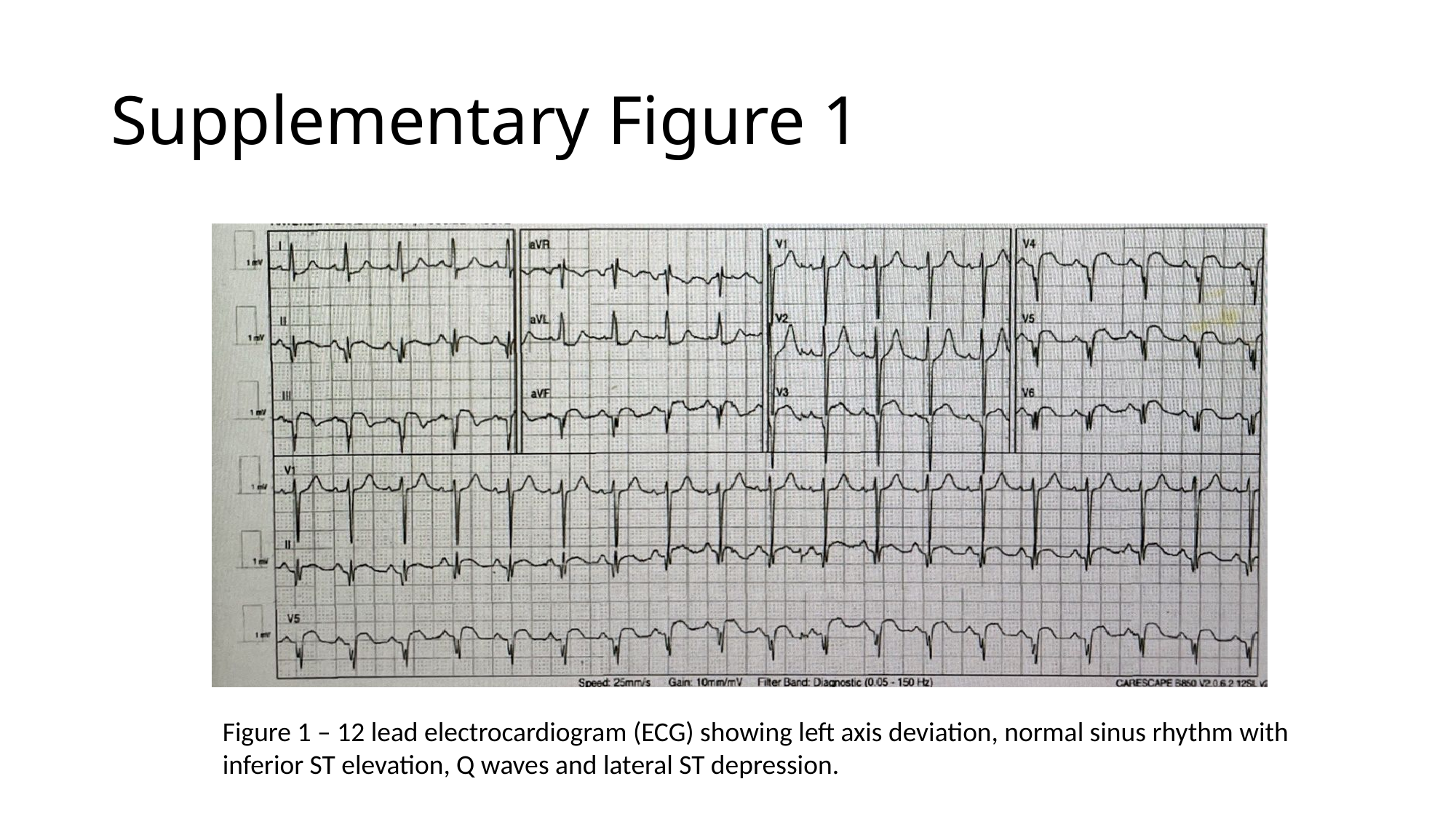

# Supplementary Figure 1
Figure 1 – 12 lead electrocardiogram (ECG) showing left axis deviation, normal sinus rhythm with inferior ST elevation, Q waves and lateral ST depression.

## Slide 3
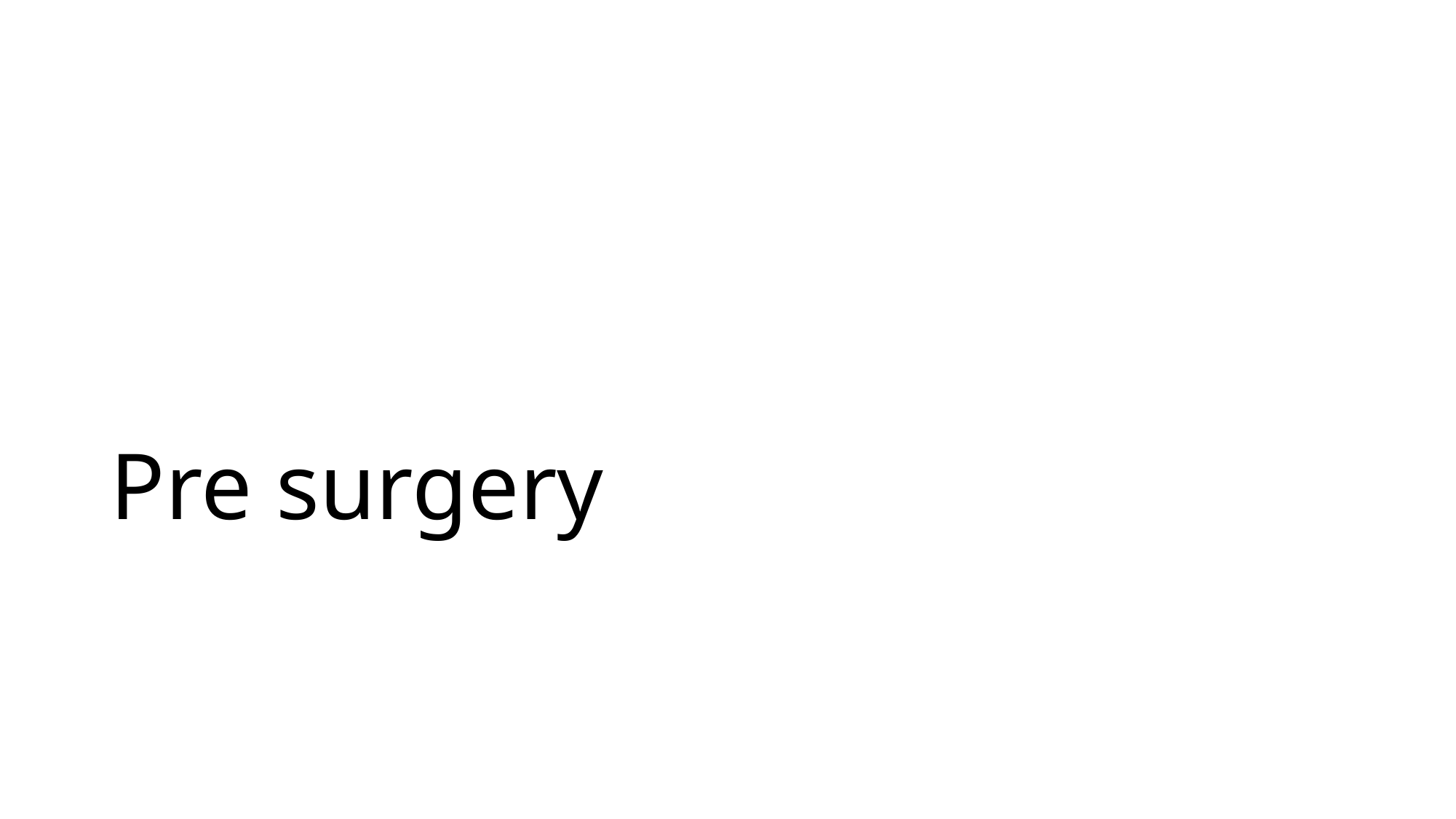

# Pre surgery

## Slide 4
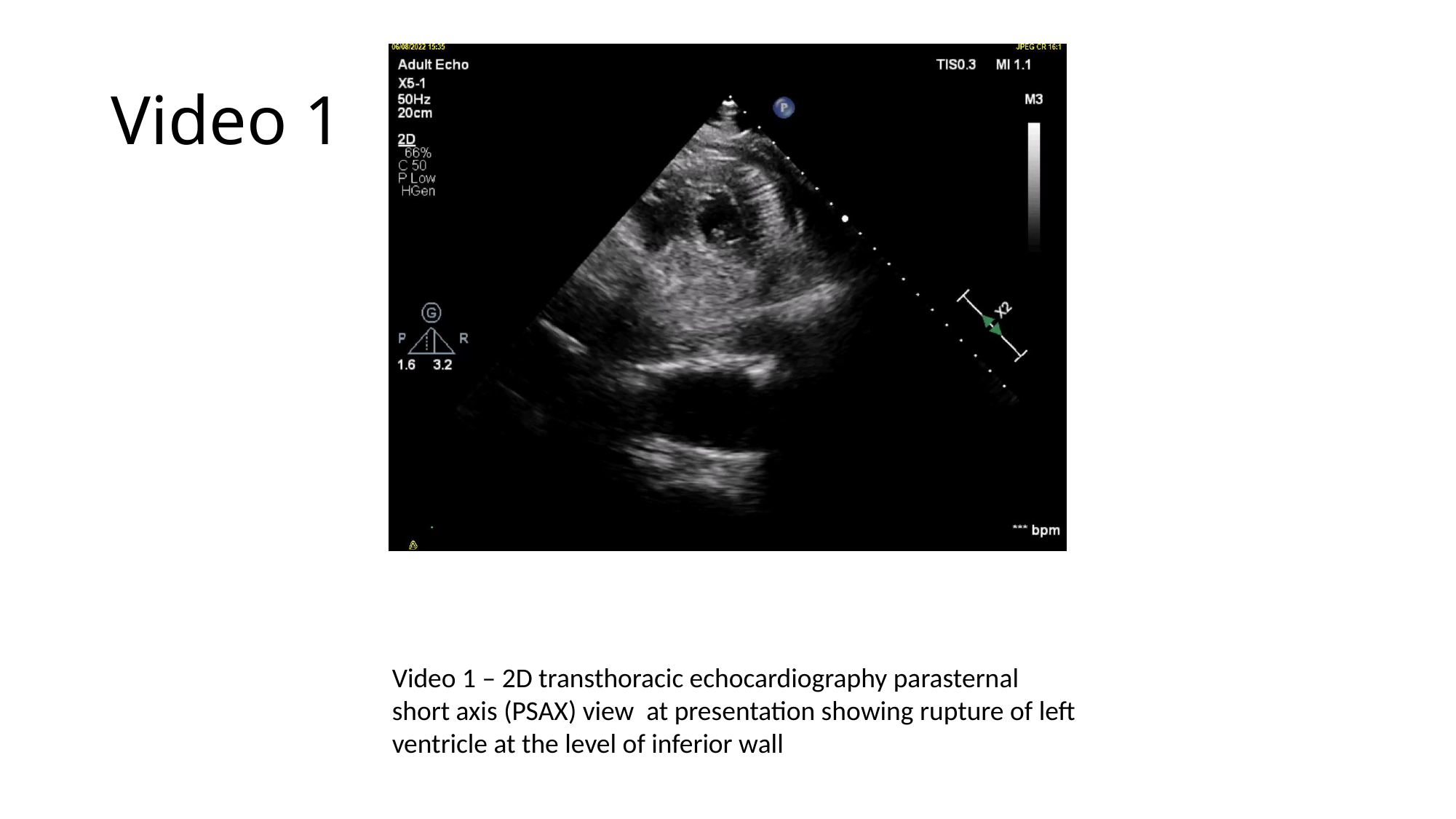

# Video 1
Video 1 – 2D transthoracic echocardiography parasternal short axis (PSAX) view at presentation showing rupture of left ventricle at the level of inferior wall

## Slide 5
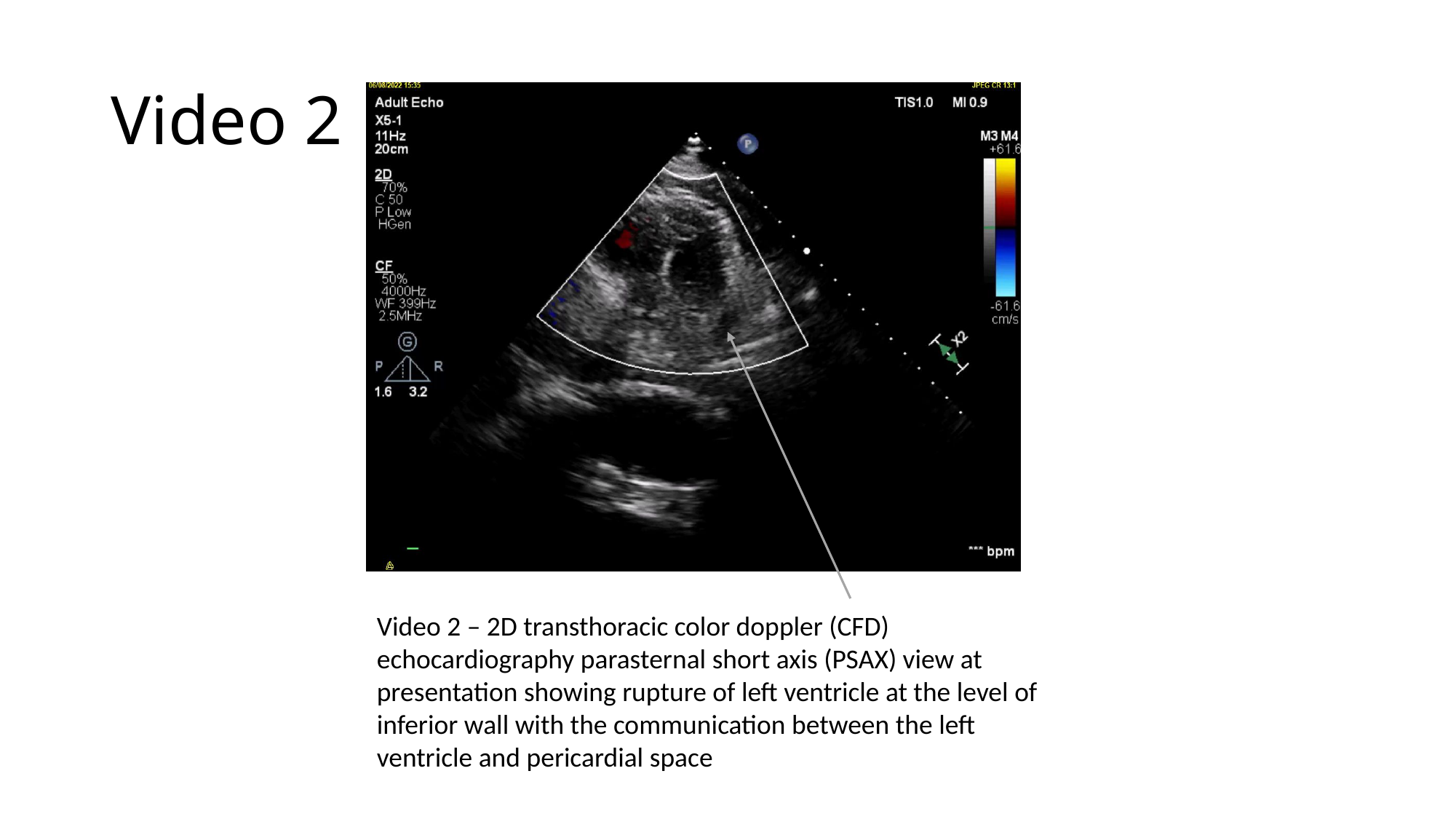

# Video 2
Video 2 – 2D transthoracic color doppler (CFD) echocardiography parasternal short axis (PSAX) view at presentation showing rupture of left ventricle at the level of inferior wall with the communication between the left ventricle and pericardial space

## Slide 6
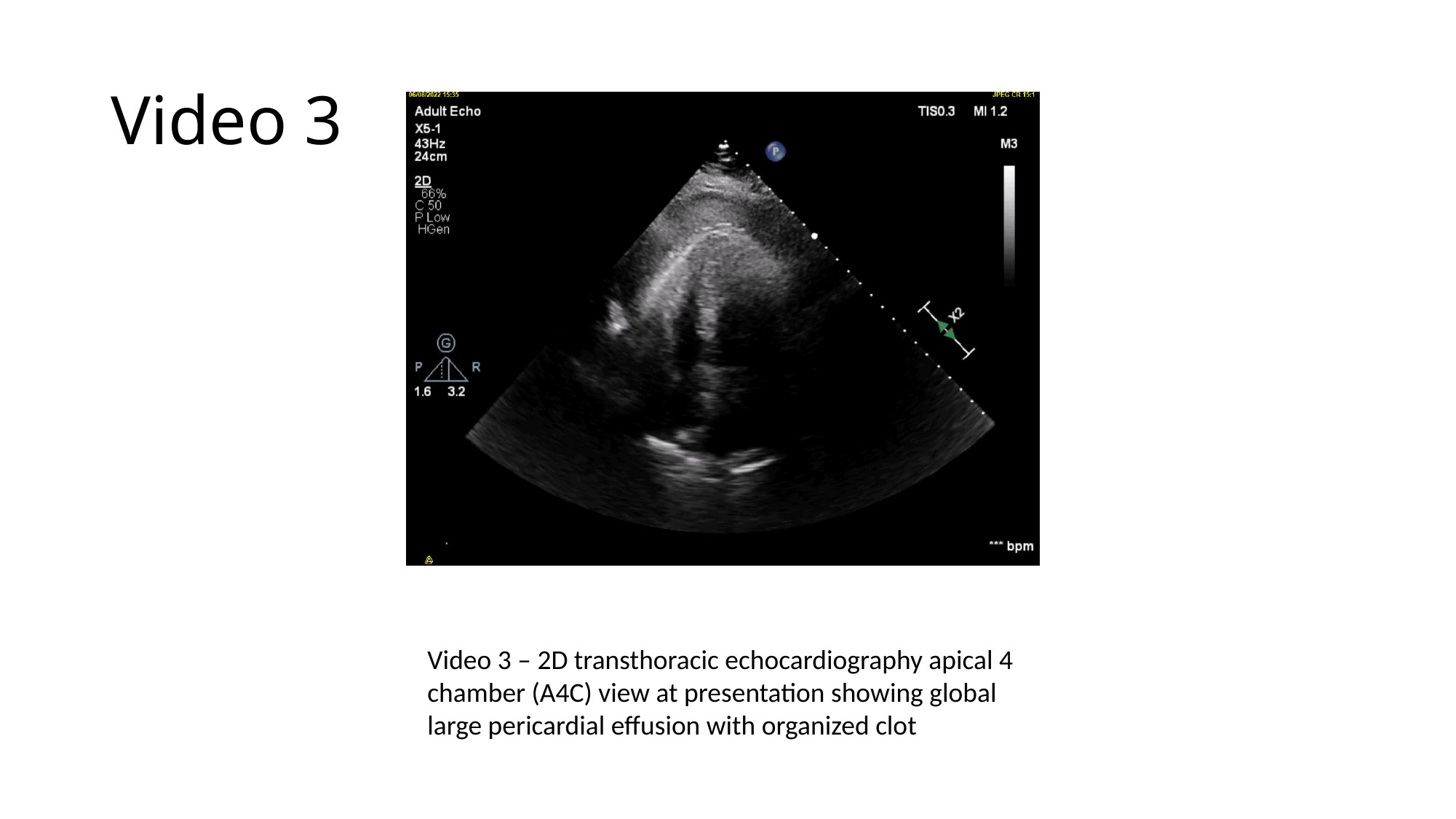

# Video 3
Video 3 – 2D transthoracic echocardiography apical 4 chamber (A4C) view at presentation showing global large pericardial effusion with organized clot

## Slide 7
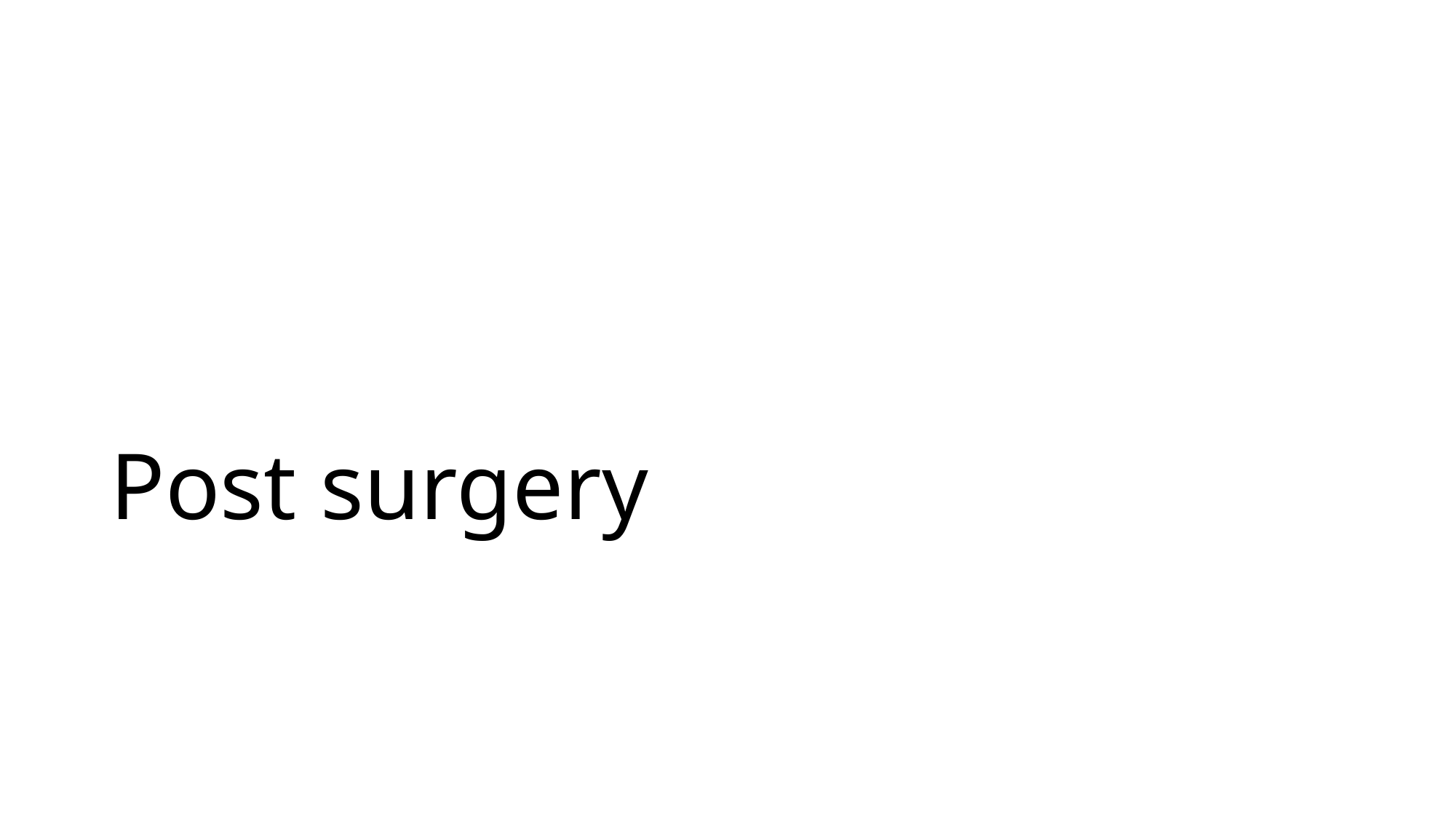

# Post surgery

## Slide 8
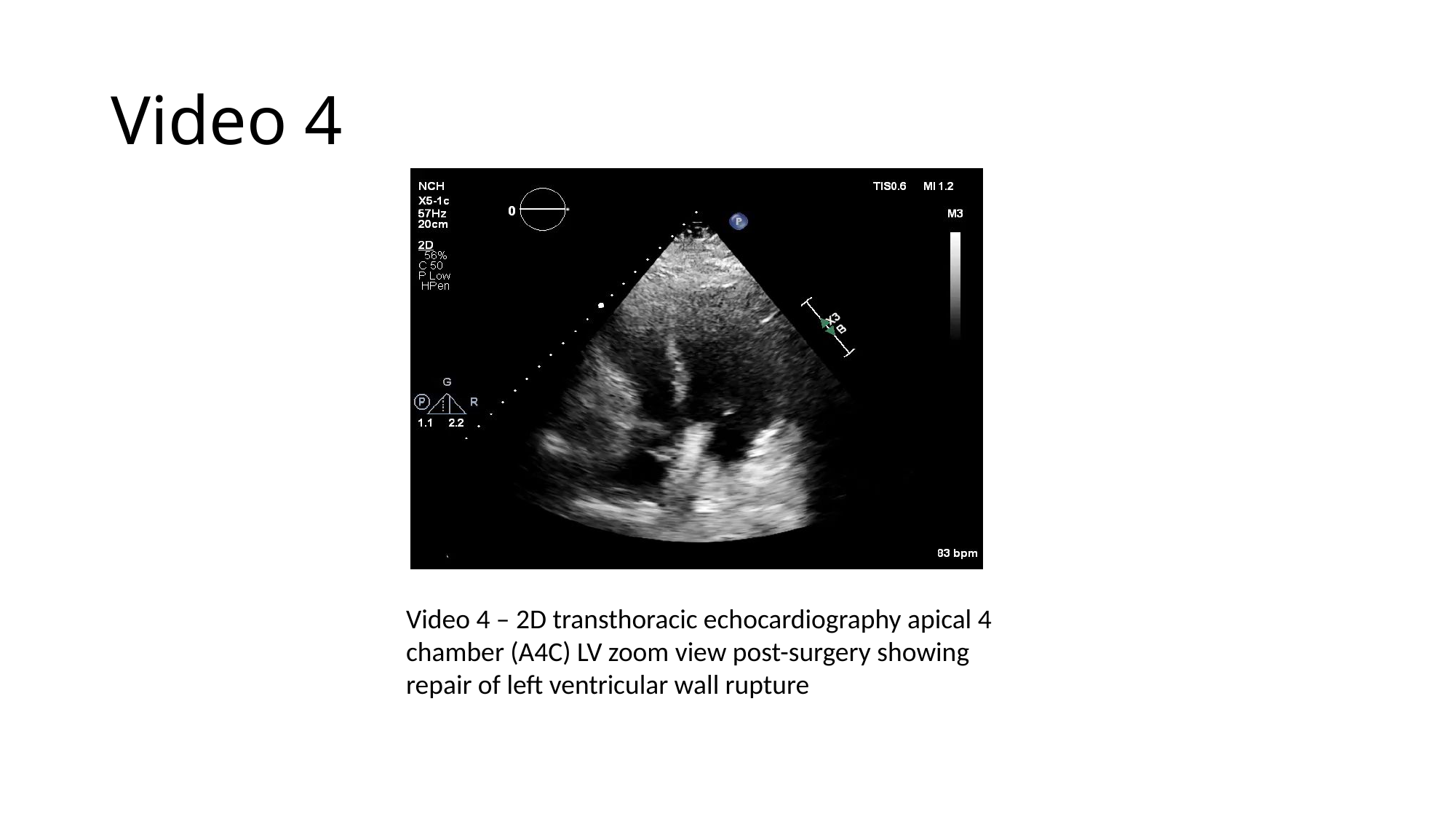

# Video 4
Video 4 – 2D transthoracic echocardiography apical 4 chamber (A4C) LV zoom view post-surgery showing repair of left ventricular wall rupture

## Slide 9
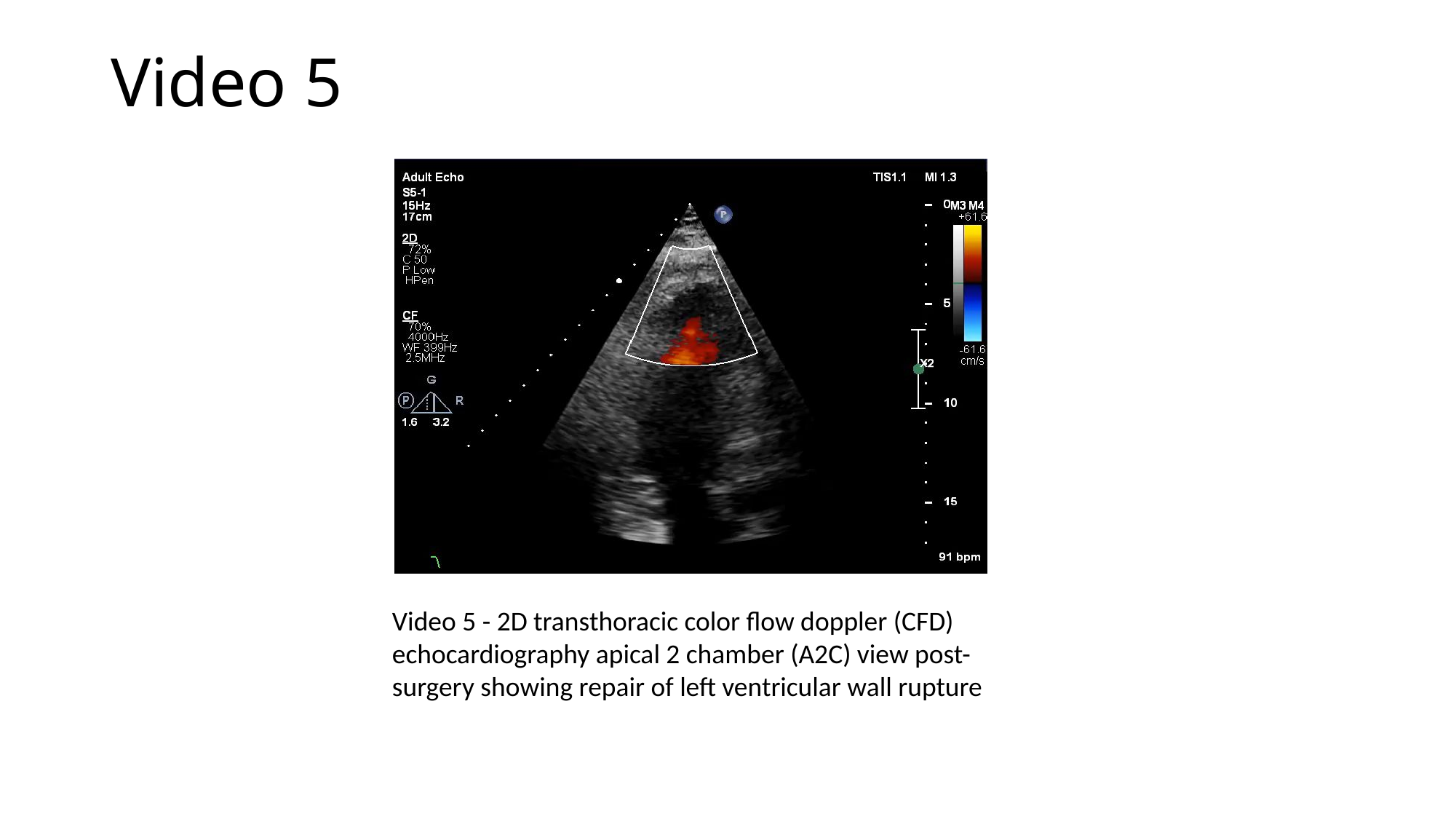

Video 5
Video 5 - 2D transthoracic color flow doppler (CFD) echocardiography apical 2 chamber (A2C) view post-surgery showing repair of left ventricular wall rupture

## Slide 10
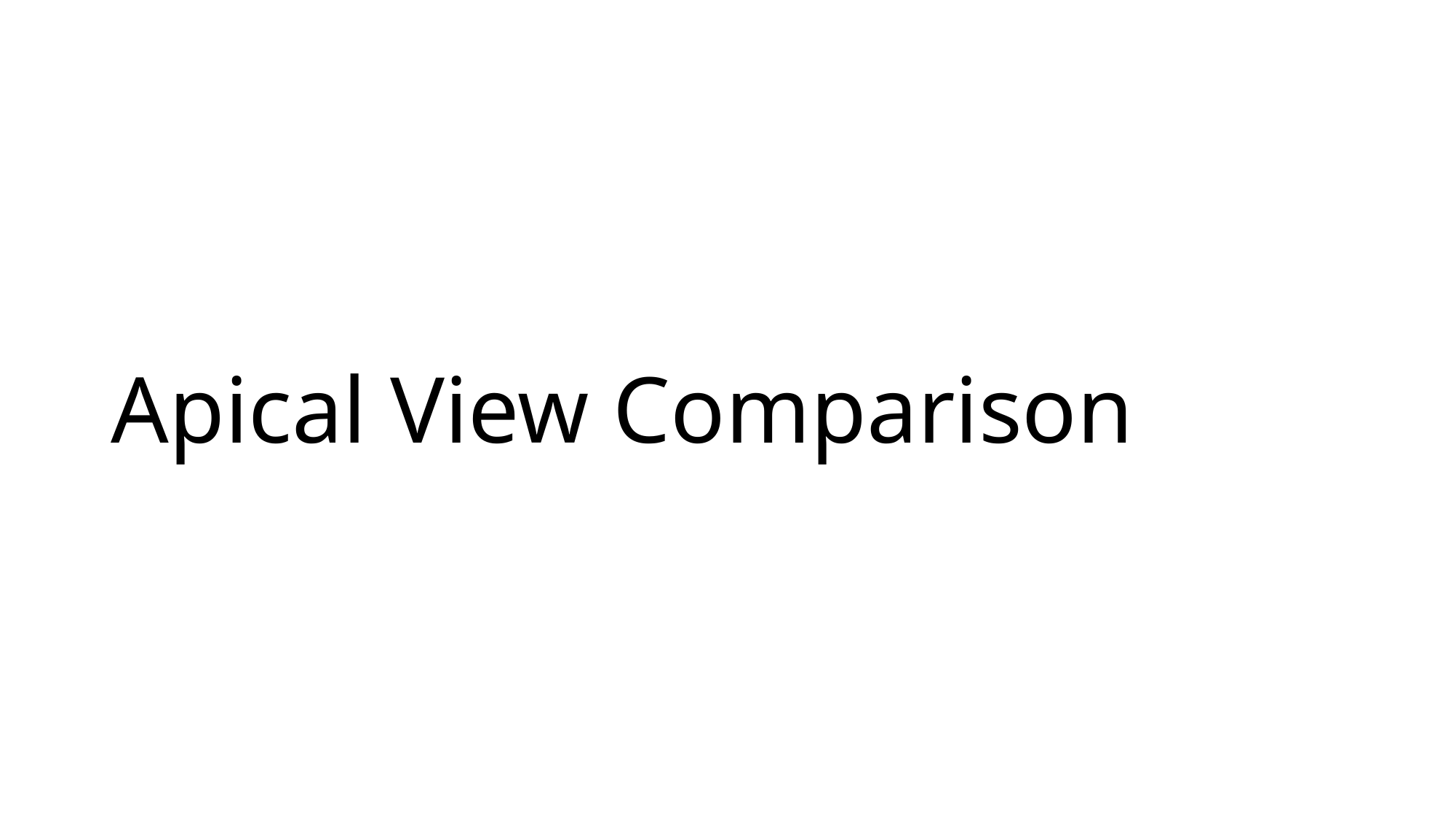

# Apical View Comparison

## Slide 11
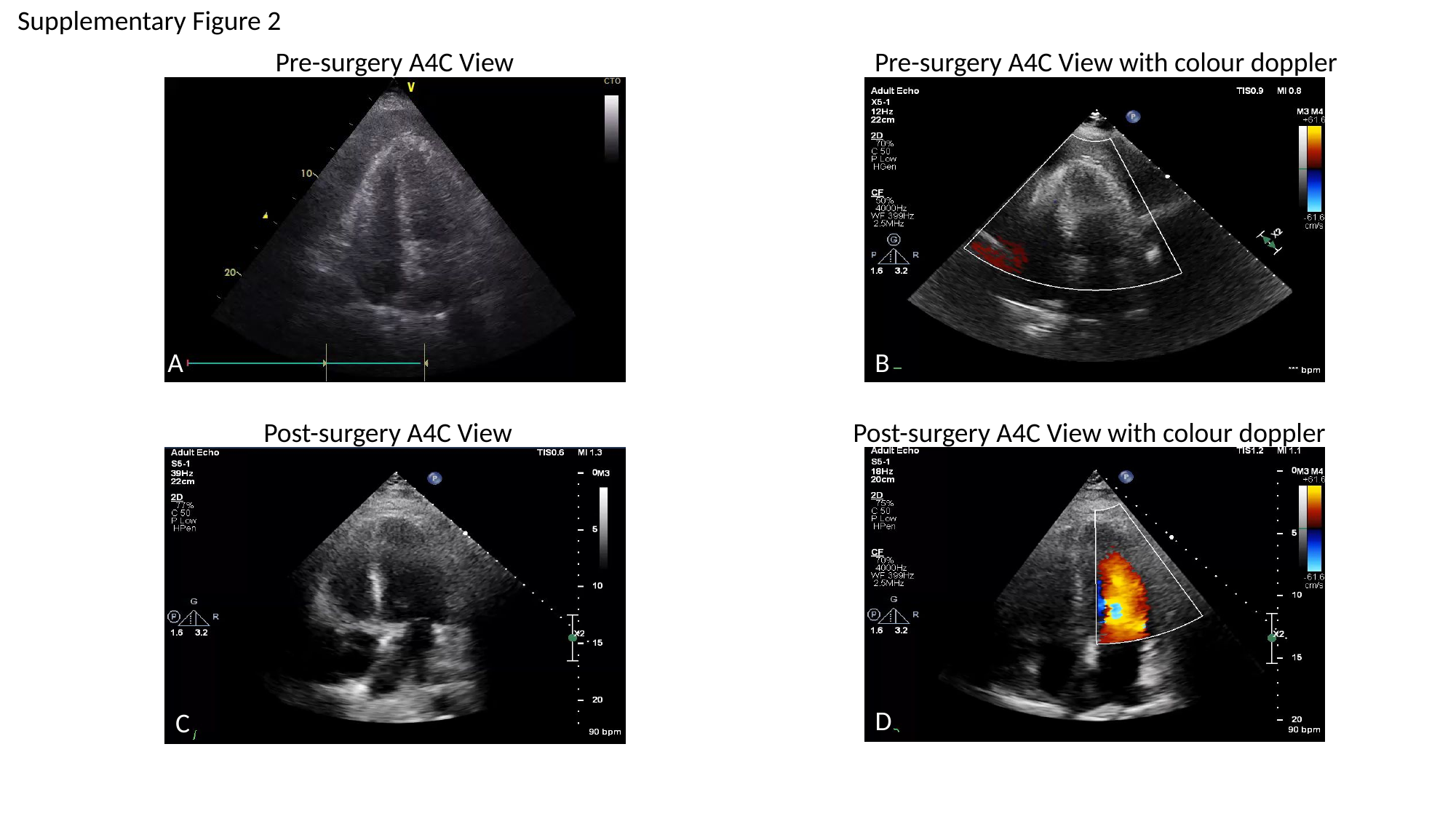

Supplementary Figure 2
Pre-surgery A4C View with colour doppler
Pre-surgery A4C View
A
B
Post-surgery A4C View
Post-surgery A4C View with colour doppler
D
C
